# LONG-TERM EFFECT OF HEALTH CARE DISCRIMINATION ON LIFE SATISFACTION OF FUNCTIONALLY LIMITED OLDER ADULTS IN ENGLAND

**DOI:** 10.1093/geroni/igae098.3255

**Published:** 2024-12-31

**Authors:** Felipe Sandoval Garrido

**Affiliations:** University of Tsukuba, Tsukuba, Ibaraki, Japan

## Abstract

Healthcare discrimination, e.g. receiving poorer service compared to others, detrimentally affects older adults, particularly those with greater care needs. In the UK, the long-term interplay of healthcare discrimination and functional limitations is under-researched, leading to potential care gaps and poorer health outcomes. This study fills this gap by examining the effects of healthcare discrimination and functional limitations on the life satisfaction of 12,690 individuals aged 60+ from the 2010-2019 English Longitudinal Study of Ageing. The 2010-11 survey assessed healthcare discrimination and functional limitations using ADL and IADL scales. Life satisfaction was measured using the Satisfaction with Life Scale, with scores from 2010-11, 2014-15, and 2018-19 analyzed via a linear mixed-effect model to account for the longitudinal design. Findings show that 2.9% (368 respondents) experienced discrimination. A preliminary bivariate logistic regression showed that those with more significant functional limitations were twice as likely to face discrimination (odds ratio: 2.010). The mixed-effect model showed a 1.580-point decrease in life satisfaction due to discrimination and a 1.134-point decrease from ADL/IADL difficulties. An interaction effect led to a larger 2.399-point drop when combining both factors, adjusted for demographic and health variables. These results highlight the intersection of healthcare discrimination and functional limitations, emphasizing the need for targeted policy and educational reforms. Incorporating these insights into training programs for healthcare professionals and establishing more inclusive, sensitive healthcare policies could significantly enhance care quality and outcomes for the elderly. This study underscores the imperative for such initiatives to better cater to the aging population’s unique needs.

